# Diseases of the Glands of the Female Urethra

**Published:** 1881-02

**Authors:** A. J. C. Skene


### Selections.

### Diseases of the Glands of the Female Urethra. By A. J. C. Skene, m.d.

Last spring I called attention to the anatomy of two important glands of the female urethra, and made some contributions to their pathology. Since that time I have had opportunities for extending my observations, and I now offer the result of my investigations to this Society. For the benefit of those who may have overlooked the original paper in the *Obstetrical Journal*, and with the view of making what follows more clear, I will quote briefly the description of the anatomy of these glands.

Upon each side near the floor of the female urethra there are two tubules large enough to admit a No. 1 probe of the French scale. They extend from the meatus urinarius upwards, from three-eighths to three-quarters of an inch, parallel with the long axis of the urethra. They are located beneath the mucous membrane in the muscular walls of the urethra. The mouths of these tubules are found upon the free surface of the mucous membrane of the urethra within the labia of the meatus urinarius.

The location of the openings is subject to slight variation, according to the condition and form of the meatus. In some subjects, especially the young and very aged, and in those in whom the meatus is small and does not project above the plane of the vestibule, the orifices are found about an eighth of an inch within the outer border of the meatus. When the mucous membrane is thickened, relaxed and slightly prolapsed, or when the meatus is everted, conditions not uncommon among those who have borne children, the openings are exposed to view upon each side of the entrance to the urethra. The upper ends of the tubules terminate in a number of divisions which branch off into the muscular walls of the urethra. These branches can be demonstrated by injecting the tubules with mercury and then laying them open.

I have called them glands because they differ in size and structure from the simple follicles found in abundance in this portion of the mucous membrane. When they were first discovered I presumed that they were mucous follicles which were accidentally of unusual size in the subject examined, but having investigated more than two hundred of them in as many different subjects, in which they were present and uniform in size and location, I became satisfied that they were worthy of a separate place in descriptive anatomy. This attention they have not heretofore received, nor have the diseases to which they are subject been referred to by pathologists. At least I have been unable to find any notice of them in the standard text-books on anatomy and gynæcology in English, French and German. It is easy to understand why these insignificant structures should have been overlooked by anatomists, or, if noticed at all, to be classed with the ordinary mucous follicles. It is only when their pathology is understood that their real importance becomes apparent.

The diseases of these glands to which I invite your attention at the present time are:

*First.*—Subacute inflammation or catarrh.

*Second.*—Gonorrhœal inflammation and its results or products.

*Third.*—Inflammation following vulvitus such as occurs in strumous children.

*Fourth.*—Tuberculosis.

The first affection named in the classification is a mild form of catarrhal inflammation which occurs in connection with subacute vaginitis such as we find accompanying ordinary uterine disease or following parturition. This condition gives the patient very little, if any, inconvenience, and readily passes unnoticed by the gynæcologist unless especially looked for. The mouths of the ducts are slightly enlarged, and sometimes surrounded by a very narrow areola of a bright-red color. By pressure upon the urethra from behind forward they discharge a white serous fluid. The cases which have come under my observation were detected while examining for other diseases, and none of them were attended with any marked symptoms. In some of them the inflammation disappeared without treatment. In others it continued without showing any tendency to increase in severity or lead to important changes of structure. It is quite possible that a non-specific vaginitis might induce a high grade of inflammation in these glands with all the pathological changes to be described hereafter, but up to the present time I have not observed any evidence that such is the case.

Gonorrhœal inflammation of these glands is of the chronic purulent variety, and in time extends from the mucous membrane of the ducts to the surrounding tissues. It does not usually attract attention until the vaginitis and urethritis has subsided.

The lesions presented differ according to the length of time which the disease has existed. When examined early there is slight swelling of the lower portion of the urethra. The mouths of the ducts are larger than normal, and the tissues around them are congested. There is tenderness to the touch, and pressure upon the urethra from above downwards causes a free purulent discharge. Sometimes it is necessary to separate the labia of the meatus in order to see the orifices of the ducts. In cases of longer standing the mouths of the ducts are brought into view by a slight prolapsus and eversion of the mucous membrane caused by swelling. The mucous membrane in the neighborhood of the ducts becomes thickened by proliferation of the areolar tissue and epithelium, presenting an irregular papillomatous appearance of a deep red color, upon the inner sides of which the orifices of the ducts appear like minute ulcers, of a yellowish gray color. The lower third of the urethra is generally thickened and indurated. The general appearance of the parts is quite like caruncle or papilloma of the meatus. In fact, inflammation of these glands has been mistaken for caruncle, at least it has been my misfortune in the past to confound the two affections, and I cannot see how others could have made a differential diagnosis, if guided by the current literature upon the subject. In a large proportion of the cases of this disease I have observed that upon the inner side of the labia minora, which rests upon the meatus, there are patches of inflammation which are caused and kept up by the purulent discharge from the glands. These circumscribed patches of inflammation sometimes extend downwards on each side of the introitus, and occasionally involve the carunculæ myrtiformes. This gives rise to much tenderness, which simulates vaginismus. The chief symptoms are extreme tenderness to the touch, great discomfort in sitting and walking, occasional sharp stinging pain, and a continual sense of heat in the parts. There is painful urination in some cases, and in others there is not. In some of the most marked cases that I have seen, this symptom was entirely absent, while in less severe forms it has been present. That peculiar difference in the history of cases I have attributed to the fact that in the well-developed forms of the disease there is considerable eversion of the lower portion of the urethra, which throws the diseased and tender portion outwards, and thereby prevents the urine from coming in contact with the irritable surfaces. Occasionally there is frequent urination, due most likely to sympathetic irritation of the bladder. The symptom which is always present, in varying degrees of severity, is tenderness. The diagnosis and treatment may be left unnoticed until the other two affections of these glands have been described.

Purulent vulvitis, which occurs in children, especially those of scrofulous diathesis, occasionally extends to these glands. When such an extension of the disease occurs, it adds its well-known rebelliousness to treatment. The original inflammation of the vulva may be relieved, but if the glands are involved, the purulent discharge from them will soon light up the disease of the external parts. From my own observations I believe that these glands rarely become involved, but when they do there is little possibility of curing the affection of the vulva until the glands are first successfully treated. There is really nothing peculiar in the clinical history of this form of disease, except its etiology, and therefore I need not dwell longer upon it, further than to say that I have seen a case of this kind, which had resisted treatment for a long time, but promptly recovered after the inflammation of the glands was detected and treated.

Tuberculosis, or tubercular inflammation of these glands, is an affection to be distinguished from the other forms of disease already considered. It occurs only in those who are of the tubercular diathesis, and may appear as a primary affection, or be developed during the progress of tubercular disease of other organs of the body. When the disease is first established it presents the same pathological appearances as have been described under the head of gonorrhœal inflammation. There is, apparently, the same purulent discharge, with redness and proliferation around the mouths of the ducts, giving the peculiar caruncular or papillomatus appearance. The only peculiar characteristics of this affection that have been observed up to the present time, are the accumulation of caseous material in the tubules, and ulceration, which occur in more advanced stages of the disease.

The ulceration takes place in the newly formed tissue in the walls and around the mouths of the tubules. These caseous concretions and ulcerations are not found in all cases. Indeed, they are rare.

There is generally urethral inflammation accompanying this condition of the glands. It sometimes begins simultaneously with the disease of the glands, and when it does not, it follows soon after. In time the bladder becomes affected, and also the kidneys. At whatever point the disease commences it increases in severity, and extends until the whole of the urinary organs are involved, unless the patient succumbs before it has completed its progress. In some cases there are polypi and papillary growths of small size found along the urethra. These, I believe, originate in inflammation of mucous follicles and papillæ of the mucous membrane.

The symptoms presented in this form of disease are the same as those found in other forms already described. From this it will be observed that the physical appearance and the symptoms are insufficient to establish a diagnosis. When there are ulcerations and caseous deposits the disease may be strongly suspected of being tubercular. Still, there is room for doubt until we find tuberculosis of other organs. This either precedes or soon follows the appearance of the disease of the glands.

In all the cases which have come under my observation the lungs were either tubercular when the patients were first seen, or became so soon after.

This affection is a source of great annoyance and suffering, and no doubt hastens the progress of the pulmonary disease with which it is generally accompanied. It has also another very important significance in the fact that it indicates the commencement of general tuberculosis of the urinary organs. The diagnosis of tubercular cystitis and urethritis has always been exceedingly difficult in the early stages of the disease. Indeed, it has been deemed impossible by most authors to distinguish ordinary cystitis from the tubercular form until the disease became developed in other organs of the body. Now that tuberculosis of these glands is understood, a valuable aid to diagnosis has been gained. Whenever an inflammation of these glands is found that cannot be traced to a former gonorrhœa or vulvitis, it is almost sure to be tubercular, and the diagnosis is placed beyond doubt if the patient has the tubercular diathesis.

I am greatly indebted to Dr. Terrillon, of Paris, for some very valuable information upon the relations of disease of these glands to tuberculosis. In the *Progrés Médical* for this year he published a very elaborate article entitled "Polypoid Excrescences of the Female Urethra, Symptomatic of Tuberculosis of the Urinary Organs," which is full of original observations of inestimable value. In comparing his observations with my own I am fully satisfied that he has mistaken tubercular inflammation, and its products, of these glands, for excrescences, in some of his cases at least. Without being aware of the presence of these glands, it is perfectly natural that he should class those vascular developments found at the meatus urinarius among the ordinary neoplasms of the urethra, just as all others have done in the past. There is every reason for believing that the excrescences which Dr. Terrillon refers to differ in their essential pathology from the ordinary polypoid growths, usually called carunculæ, which are found in the urethra, and are associated with tuberculosis. And as the history of his cases coincides with the history of the cases of tuberculosis of these glands which I have seen, I am compelled to believe that he has not fully comprehended the true pathology of this affection. He has, however, clearly shown the relation of this affection to tuberculosis of the urinary organs, and that alone is worthy of the highest honor.

Dr. Terrillon's article is too long to be given in full, but a few condensed extracts will show his views upon the subject. His description of the symptoms and the general appearance of the parts affected are so much more complete than my own that I prefer to give them in full:

"The fungoid growths show themselves usually at the surface of the urethral orifice. They are projecting and pedunculate. Seldom isolated, they form most frequently a wreath, more or less regular, around the orifice of the meatus. In very aggravated cases they are united into a mass and then form a real projecting tumor with a fringed aspect, of a lively red. In the center of the tumor is easily to be found the orifice of the urethra masked by those papillary growths. The symptoms of fungoid excrescences of the urethra accompanying tuberculosis of that organ and the bladder includes the observation of two distinct parts: First, the study of the growths themselves and the character of them. Second, all the phenomena to be found in cystitis and tubercular urethritis. Sometimes the symptoms of the two lesions are found together; sometimes, on the contrary, they exist singly up to a certain period of the disease. One of the special symptoms of this affection is the exquisite tenderness of which these fungoids are possessed. The least touch, the least rubbing, the passage of urine suffices to cause the most extensive pain which renders life insupportable. This hyperæsthesia, which may extend to the neighboring parts, causes at the sides of the orifice of the vulva symptoms of the most acute vaginitis. These are the ordinary symptoms of fungoid growths when existing externally." The author at this point refers to excrescences found within the urethra as being of the same nature as those found at the meatus. He makes no distinction between the two forms of disease. There is, however, a difference worthy of notice. Excrescences found within the urethra are usually cystic polypi or enlarged papillæ of the mucous membrane, conditions which may exist independently of tuberculosis. I infer from some other statements made in his writings that the granular urethritis—as we are in the habit of calling it—is generally secondary to the disease of the urethral glands. The views of this author in regard to the order of development of urethritis, cystitis, and finally tuberculosis of the lungs, are set forth in the following:

"Sometimes, at the time of their appearance, these fungoids appear to be altogether isolated from all other serious lesions. Yet they seem to precede tuberculization, or soon take a rapid course in developing granulations in the urethra. In other cases these growths may appear some time after the symptoms of tuberculization have been established." The cases recorded by Dr. Terrillon, and also those which have come under my own observation, show that, as a rule, this disease of the urethra precedes the appearance of tuberculosis in other organs of the body, such as the lungs. It also is one of the first lesions observed in tuberculosis of the urinary organs. The following is from Dr. Terrillon's paper on this subject:

"Now comes up the important question, whether these polypi of the mucous membrane should be considered as a primary or an idiopathic lesion, and I think that it can be solved in the following manner: These polypi are most assuredly the result of chronic inflammation and an irritation of the mucous membrane. Now, development of tubercular granulations within the mucous membrane is at first the cause of irritation, before any changes in the urine; ulceration does not occur until after a sufficient length of time. With one of our patients the first irritation induced the formation of polypi, and the common painful symptoms followed. Their extirpation gave relief, but that lasted only up to the time when urethro-vesical ulceration occurred. It will be observed that in this case the affection began in the urethra and extended to the bladder, and also secondarily involved the left kidney (ascending tuberculosis), causing finally change in the urine, with the free formation of pus. I therefore do not hesitate to maintain that the fungoid polypi are the result of tubercular irritation of the mucous membrane of the urethra, which gives rise to the very serious symptoms which occur in the early stages of the disease. Without them urinary tuberculosis would not give rise to those striking symptoms until after a sufficient length of time, when the ulcerations appear in other organs. An analogous phenomenon, which is observed in the larynx, should be mentioned here. We know, as a matter of fact, that the tuberculization of the larynx does not only occasion ulceration, but also polypoid growths. There is produced at the expense of the ulcerated mucous membrane an hypertrophy and proliferation, in the form of cauliflower excrescences or cock's-comb growths, a species of polypi smaller or larger, by which the glottis might be more or less obliterated. It will therefore be admitted that there is a resemblance between laryngeal excrescences and those found in the urethra of women.

The polypoid excrescences of the female urethra show, from an etiological point of view, to be of two distinct varieties. The first variety is idiopathic, and may be recognized by a slight irritation. The prognosis is good; extirpation in these cases gives a rapid cure. This is the most frequent variety. The second kind, although they give the same outward appearances as the first variety, are on the contrary accompanied from the outset by urethritis and tubercular cystitis, of which variety these lesions constitute important symptoms.

When I found inflammation of these glands associated with tuberculosis of other organs, it occurred to me that the disease of the glands might be of the same nature, or tubercular, but I am indebted to the writings of Dr. Terrillon for the full knowledge of the pathological relations of the affection of these glands to tuberculosis of the other urinary organs. We have studied the subject from different standpoints, and the combined results of our labors cover the ground pretty thoroughly. While he has clearly settled the relation of these excrescences to tuberculosis of the urinary organs, I have satisfied myself that these new growths are but the products of a tubercular inflammation of the urethral glands, the existence of which were, I presume, unknown to him. The treatment of the various forms of inflammation of those glands may all be discussed at the same time.

It is settled upon the best evidence that when these glands become inflamed there is no natural tendency to their recovery. Those who have read the history of my first published case will remember that I employed all the recognized treatment for caruncle, but at the end of a year my patient was no better. Dr. Terrillon has had a similar experience. On this point he says: "A characteristic more important, and to which I desire to call especial attention, because it indicates well in my opinion the consecutive development of these excrescences, is their tenacity and the facility with which they recur. Really, one can see in the observations (meaning his cases) in which continued surgical intervention has been practiced, it brought about either no relief or only a momentary amelioration."

This experience in the treatment of these excrescences is proof positive to me that they originated in inflammation of the urethral gland. My own observations have given the same results, showing clearly that these growths cannot be arrested until the inflammation of the glands which causes them is eradicated. It is otherwise with polypoid excrescences due to other causes. If they are completely removed they do not return as a rule.

The treatment which I employed at first was to inject the tubules with the ordinary solutions used in the treatment of inflammation of mucous membranes, using for the purpose a hypodermic syringe, with the point of the middle rounded off. This method I found useful, but very tedious. It then occurred to me that laying open the tubules their whole length and keeping them open would prevent the purulent accumulation (which acts so effectually in keeping up the inflammation) and also bring the affected parts within easy reach of the necessary treatment. This method was suggested in my paper published last winter, and since then I have tried the method in quite a number of cases and found it entirely satisfactory. In the majority of cases it is all that is required to effect a complete cure. The method of operating is as follows: The patient is placed upon the left side, and a Sims speculum used to keep the labia apart and retract the perineum. This brings the parts well into view, and within easy reach of the operator.

The position and depth of the tubules having been first ascertained, the probe-pointed blade of a very fine scissors is then introduced, and the posterior wall divided its whole length. To prevent the parts from reuniting, a small piece of cotton, saturated with persulphate of iron, should be packed in between the divided edges. Brushing the surfaces over with the iron, without using the cotton, will answer, although less certainly, to prevent reuniting. Very little after-treatment is required. In the majority of cases recovery follows the operation of laying open the canals. Sometimes the inflammation lingers in a modified form, but yields to a few applications of nitrate of silver or sulphate of zinc. In several cases in which the excrescences were abundant, they remained after the operation, although very much reduced in size. An application of nitric acid destroyed them, and they have not shown the least disposition to return.

